# Perceptions toward and practices regarding antibiotic stewardship and use among physicians at tertiary-care public hospitals in Bangladesh

**DOI:** 10.1017/ash.2022.56

**Published:** 2022-05-16

**Authors:** Shariful Amin Sumon, Saiful Islam, Golam Dostogir Harun

## Abstract

**Background:** The emergence of antimicrobial resistance (AMR) is affecting public health management in developing countries, including Bangladesh. Irrational and inappropriate use of antibiotics in healthcare settings has led to widespread drug resistance. To optimize antimicrobial usage to combat AMR and enhance infection treatment, the competence of healthcare workers in antibiotic prescribing is indispensable. We sought to determine the perceptions about antimicrobial resistance, antibiotic stewardship programs (ASPs), and antibiotic prescribing approaches among physicians at tertiary-care public hospitals in Bangladesh. **Methods:** From September to December 2020, we conducted a mixed-methods study in 9 tertiary-care public hospitals. Using a self-administered, semistructured questionnaire, we collected data on antibiotic stewardship and prescribing practices from 452 on-duty physicians. In addition, we conducted 16 key-informant interviews to explore AMR perceptions and determinants. **Results:** Only 43.8% of physicians were aware of the ASP, and none of the hospitals had any ASP initiative in place. Most of the participants (70.6%) recommended tailored training and antibiotic prescribing guidelines (63.7%) for effective ASP initiatives. More than half of the physicians (54.7%) preferred to receive regular monitoring and feedback on their routine antibiotic prescriptions from the stewardship program. In terms of the antibiotic prescribing approach, only 25.9% of physicians relied on microbiology lab findings, whereas 69.5% routinely employed oral or intravenous antibiotics. Also, 40.0% of physicians considered the patient’s ability to afford the antibiotic cost when recommending antibiotics. The qualitative investigation identified the use of broad-spectrum antibiotics, absence of guidelines, and inadequate laboratory support as factors contributing to AMR in the healthcare setting. Self-medication, over-the-counter dispensing, and patients’ economic instability to complete the dosage were also attributed to the irrational use of antibiotics. As a priority step, physicians advocated for intensive training on antibiotic advising, mass awareness campaigns on safe antibiotic usage and dispensing, and a restriction on the widespread sale of antibiotics from pharmacies. **Conclusions:** Despite favorable perceptions, the fundamental understanding of physicians regarding ASPs and rational prescribing of antibiotics needs to be improved through context-specific educational interventions and capacity building. In addition, a coherent and comprehensive policy is required for the development and implementation of antibiotic usage guidelines along with integrated ASP initiatives to combat AMR.

**Funding:** None

**Disclosures:** None

